# Statistical Machine Learning Methods to Handle Missing PHQ-8 Score – Assuming Missing at Random

**DOI:** 10.1192/bjo.2024.440

**Published:** 2024-08-01

**Authors:** Khalid Suliman, Mitha Al Balushi, Hannah Holliday, Manal Alblooshi, Amar Ahmad

**Affiliations:** 1 Public Health Research Center, New York University in Abu Dhabi, Abu Dhabi, UAE; 2 New York University, New York, USA

## Abstract

**Aims:**

Missing data is a challenge that most researchers encounter. It is a concern that continues to be analyzed and addressed for solutions. Missing data occurs when there is no data stored for certain variables relating to participants. In health surveys, when participants answer in the form of “I don't know” or “I'd prefer not to answer”, these responses can, in many cases, be categorized as missing data responses from a participant in a specific category or question.

The eight-item Patient Health Questionnaire (PHQ-8) is an essential tool in healthcare and clinical settings to assess an individual's mental health, specifically related to symptoms of depression. The items are scored on a scale from 0 to 3 with the total score obtained by summing the scores for each item. Higher PHQ-8 scores indicate the presence of depressive symptoms.

We used empirical data from a previous study on depression symptoms in patients with coronary heart disease to study the effect of considering the answers “I do not know” and “I prefer not to answer” as missing values when estimating the percentage of depression using PHQ-8. Moreover, we studied the effect of the complete case analysis and multiple imputation on parameter estimates and confidence intervals. The outcome of this study aims to shed light on the development of missing data procedural knowledge and provide methodological support for public health decision-making when data with missing values are collected.

Furthermore, this study aims to prevent the exclusion of missing data rather than to generate data.

**Methods:**

A simulation study with 1000 replicates was performed. Four common statistical machine learning methods for handling missing values were included in this study. These are K-Nearest Neighbor (KNN), K-Means, Classification and Regression Trees (CART), and Random Forest (RF) imputations. Five clusters were used for KNN and K-mean. Likewise, five multiple imputations were used for the CART and RF methods. The simulation was based on publicly available data with available PHQ-8 data for 1096 subjects. In the simulation study and for each replication, multivariate missing values were generated using the missing-at-random (MAR) assumption with 10%, 20%, 30%, 40%, and 50% proportions of missingness. The percent of depression was calculated using the PHQ-8 questionnaire and a comparison was made between estimated actual depression, complete-case analysis, KNN, Kmean, RF, and CART, respectively.

**Results:**

The Median age of the subjects was 69 (interquartile range: 61–67) and more males (72.9%) than females were included in the data. The estimated actual depression was 16.8, whereas the estimated percentage of depression varies between 6.9–13.5, 16.2–16.7, 16.3–16.7, 16.6–16.7 and 16.7–16.8 for the complete case, KNN, Kmean, RF and CART respectively.

**Conclusion:**

The results of this simulation study show that missing PHQ-8 data are best handled by applying multiple imputations based on CART or RF. However, using K-Means or KNN leads to a good estimate of the true percentage of depression. Furthermore, the results of this simulation study show that complete-case analysis leads to biased estimates of the true percentage of depression. Nevertheless, further investigation is needed to address the problem of missing PHQ-8 data under the assumption of missing not at random.

